# 

**Published:** 1892-10-08

**Authors:** 


[Registered at the General Post Office as a Newspaper for transmission in the United Kingdom and Abroad.]

WITH EXTRA NURSING SUPPLEMENT

Per Annum, 8s. 6d., post free.

Monthly Parts, 6d.; post free, 8d.

Ditto per Annum, 8s., post free.

### THE HOSPITAL

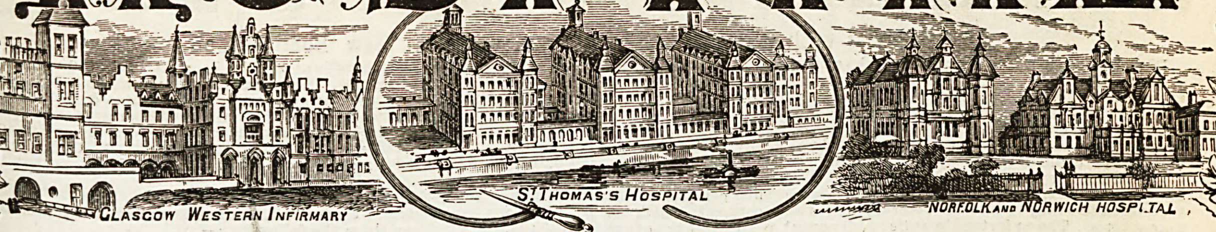

WITH EXTRA NURSING SUPPLEMENT.

No. 315. Vol. XIII.] SATURDAY, OCTOBER 8th, 1892. [TWOPENCE.

